# Cost-Effectiveness of AI for Risk-Stratified Breast Cancer Screening

**DOI:** 10.1001/jamanetworkopen.2024.31715

**Published:** 2024-09-05

**Authors:** Harry Hill, Cristina Roadevin, Stephen Duffy, Olena Mandrik, Adam Brentnall

**Affiliations:** 1 School of Medicine and Population Health, University of Sheffield, Sheffield, United Kingdom; 2 Nottingham Clinical Trials Unit, University of Nottingham, Nottingham, United Kingdom; 3 Wolfson Institute of Population Health, Queen Mary University of London, London, United Kingdom

## Abstract

**Importance:**

Previous research has shown good discrimination of short-term risk using an artificial intelligence (AI) risk prediction model (Mirai). However, no studies have been undertaken to evaluate whether this might translate into economic gains.

**Objective:**

To assess the cost-effectiveness of incorporating risk-stratified screening using a breast cancer AI model into the United Kingdom (UK) National Breast Cancer Screening Program.

**Design, Setting, and Participants:**

This study, conducted from January 1, 2023, to January 31, 2024, involved the development of a decision analytical model to estimate health-related quality of life, cancer survival rates, and costs over the lifetime of the female population eligible for screening. The analysis took a UK payer perspective, and the simulated cohort consisted of women aged 50 to 70 years at screening.

**Exposures:**

Mammography screening at 1 to 6 yearly screening intervals based on breast cancer risk and standard care (screening every 3 years).

**Main Outcomes and Measures:**

Incremental net monetary benefit based on quality-adjusted life-years (QALYs) and National Health Service (NHS) costs (given in pounds sterling; to convert to US dollars, multiply by 1.28).

**Results:**

Artificial intelligence–based risk-stratified programs were estimated to be cost-saving and increase QALYs compared with the current screening program. A screening schedule of every 6 years for lowest-risk individuals, biannually and triennially for those below and above average risk, respectively, and annually for those at highest risk was estimated to give yearly net monetary benefits within the NHS of approximately £60.4 (US $77.3) million and £85.3 (US $109.2) million, with QALY values set at £20 000 (US $25 600) and £30 000 (US $38 400), respectively. Even in scenarios where decision-makers hesitate to allocate additional NHS resources toward screening, implementing the proposed strategies at a QALY value of £1 (US $1.28) was estimated to generate a yearly monetary benefit of approximately £10.6 (US $13.6) million.

**Conclusions and Relevance:**

In this decision analytical model study of integrating risk-stratified screening with a breast cancer AI model into the UK National Breast Cancer Screening Program, risk-stratified screening was likely to be cost-effective, yielding added health benefits at reduced costs. These results are particularly relevant for health care settings where resources are under pressure. New studies to prospectively evaluate AI-guided screening appear warranted.

## Key Points

**Question:**

What is the cost-effectiveness of artificial intelligence (AI)–guided risk-stratified mammography screening intervals in the United Kingdom National Breast Cancer Screening Program?

**Findings:**

This decision analytical model evaluated 4 AI-based risk-stratified screening interval regimens in comparison with the current United Kingdom screening program. The net monetary benefit of introducing the optimal regimen ranged from approximately £60.4 million (US $77.3 million) to £85.3 million (US $109.2 million).

**Meaning:**

These results suggest that AI-based risk stratified breast cancer screening programs may be more cost-effective screening programs, providing additional health benefits with fewer resources than universal screening intervals for all.

## Teaser

This decision analytical modeling study assesses the potential cost-effectiveness of integrating risk-stratified screening using artificial intelligence (AI) into the United Kingdom National Breast Cancer Screening Program for women aged 50 to 70 years.

## Introduction

Early detection of breast cancer is a top priority for the United Kingdom (UK) National Health Service (NHS).^1^ The NHS largely uses an age-based screening strategy,^2^ inviting women aged 50 to 70 years for mammography screening triennially; most other countries with a breast screening program adopt a biennial program.^3^ This one-size-fits-all approach might be improved by tailoring screening so that those at highest risk receive the greatest intensity of screening (risk-based screening).

The NHS is considering the integration of artificial intelligence (AI) and machine learning into mammogram interpretation for breast screening in the future.^1,4^ AI is currently not used in NHS breast screening appointments in the UK due to a lack of high-quality prospective studies,^5^ but emerging prospective data are promising.^6,7^

In parallel with AI for cancer detection, AI for risk prediction has been proposed. One such model (Mirai) interprets data automatically generated from mammogram screenings, without the need for data collection through questionnaires.^8,9^ The AI model offers an immediate estimation of an individual’s short-term risk of cancer incidence following a mammogram with negative findings. The AI model is open source and freely available for research and is arguably the best (retrospectively) validated AI model for short-term breast cancer risk assessment. One analysis included an external validation across 7 hospitals spanning 3 continents,^8^ and overall, evidence suggests that in the short term, it attains a higher area under the receiving operating characteristics curve than classical risk models or breast density.^9,10,11^

While the performance of the AI model for risk assessment is highly promising, one area that has not been rigorously evaluated is whether using the model to guide screening could offer value for money. Our aim was therefore to assess the potential cost-effectiveness of integrating risk-stratified screening using the model into the UK National Breast Cancer Screening Program, through a health economic model. We considered risk-based screening strategies that would be expected to require the same number of screens as the current triennial program, assuming perfect adherence.^12^ In practice, strategies that involve extended screening intervals for a larger proportion of the population than those who receive more frequent screening might require fewer screens overall.

Specific risk-stratified breast cancer screening regimens (RSBCRs), determined by risk thresholds, were based on recent work that used a simplified deterministic model to evaluate potential effectiveness.^12^ These RSBCRs involve screening intervals aligned with corresponding risk thresholds tailored for the AI model chosen for this research. In this report, we developed an economic model to assess the cost-effectiveness of 4 AI-based screening strategies in comparison with the current screening program. The economic model was then used to estimate health-related quality of life, survival, and NHS costs (reported in pounds sterling; to convert to US dollars, multiply by 1.28) over the lifetime of the female population eligible for screening in the UK. Results from our analysis might inform future prospective evaluations of AI-guided screening.

## Methods

This study was conducted from January 1, 2023, to January 31, 2024. We followed the Consolidated Health Economic Evaluation Reporting Standards (CHEERS) reporting guideline and the National Institute for Health and Care Excellence methods of technology appraisal manual.^13^ We developed a discrete event simulation^14^ model in R, version 4.2.2,^15^ using the simmer’ package (R Project for Statistical Computing)^16^ to accommodate individual attributes that evolve over time within the simulation such as breast cancer risk.^14^ The work was funded by Cancer Research UK and the policy research unit in Economic Evaluation of Health and Care Interventions. Because the data are drawn from publicly available sources, ethics approval is not required for decision modeling in the UK.

### Strategies and Models

The following 4 strategies^12^ were distinguished by screening intervals in years for groups categorized by a 3-year risk score (RS):

1. One year (RS, ≥2.57%), 2 years (RS, 1.32%-2.57%), 3 years (RS, 1.22%-1.32%), or 6 years (RS, ≤1.22%)
2. One year (RS, ≥2.72%), 3 years (RS, 1.23%-2.72%), or 6 years (RS, ≤1.23%)
3. Two years (RS, ≥2.79%), 3 years (RS, 1.35%-2.79%), or 6 years (RS, ≤1.35%)
4. One year (RS, ≥2.20%), 3 years (RS, 1.23%-2.20%), or 4 years (RS, ≤1.23%).

The model focused on 5 dynamic processes: (1) Women transition between risk groups based on screen attendance and AI-model–assessed risk scores. (2) Mammogram accuracy is based on breast density, which changes as women age. (3) Attendance patterns to screening appointments are influenced by age and attendance history. (4) Attendance history and time intervals between screenings impact cancer stage. (5) Cancer prognosis is influenced by age, where it was identified (at regular screening or not), and attendance history, with worse outcomes for women who have not attended breast screenings.

### Structure and Model Events

**Figure 1.**
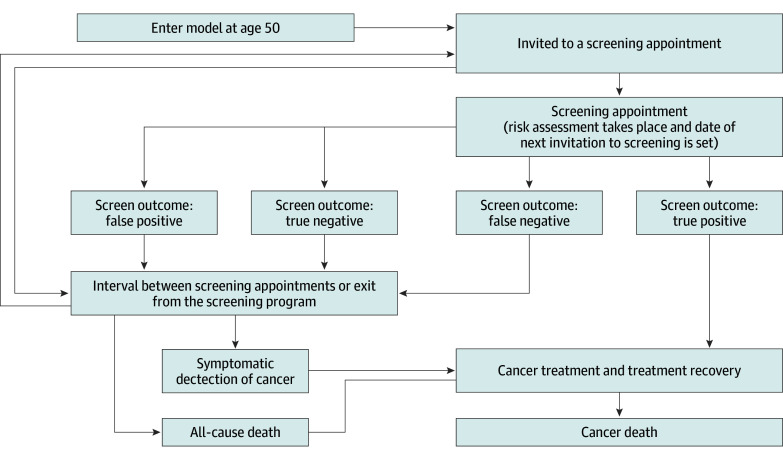
Model Structure Arrows show potential event transitions.

Figure 1 depicts the model structure, illustrating the sequence of clinical events and potential pathways from initiation to screening, cancer detection, and mortality. Women enter the model at the first screening invitation at 50 years of age, with subsequent invitations occurring at regular intervals until 70 years of age. Each screening appointment includes an updated AI risk assessment, determining the timing of the next screen invitation. If a woman misses her initial screening, invitations are sent every 3 years thereafter. The model ends when individuals reach cancer-related mortality or mortality from all other causes.

At entry, women are assigned predetermined ages of noncancer mortality and have a chance of developing breast cancer up to 74 years of age, sourced from Office for National Statistics life expectancy tables (2018-2020)^17^ and NHS breast cancer registry data.^18^ The age at which symptomatic cancer is detected in a primary care setting is sampled from 2020 incidence data,^18^ adjusted for age-related lead time in the UK screening program using overdiagnosis estimates.^19^ Screening may identify cancer at an earlier age than this determined age of symptomatic cancer. Data on attendance from 2018 to 2019,^20^ stratified by age and screening history, were used to calculate attendance probability to a screening invitation. The NHS incurs a cost of £14.52 (US $18.59)^21^ for each screening invitation (details of screening costs are in eTable 2 in Supplement 1). Screening invitations end at 70 years of age or the nearest subsequent year if screening is not scheduled at 70 years of age. For instance, in simulating the current program with screenings every 3 years, a woman attending screening at 68 years of age would receive her last invitation at 71 years of age, after which any developing cancers would be detected symptomatically.

#### Model Event: Screening Appointment

Attending women undergo a mammogram; if cancer is detected, further assessments are conducted. Breast density and AI model scores are from UK women aged 46 to 74 years and applied based on age and cancer presence.^11^ Details are available in eMethods 1 in Supplement 1.

In the model, a woman’s age for potential tumor detection is set before her first screening and remains constant throughout her life. Screening detection ages are calculated by subtracting the tumor presence period from the age at which symptomatic cancer is detected.^22^ The tumor presence period is derived from national breast screening program data and is based on age, with mean durations ranging from approximately 6 years at 35 years of age to 8 years at 85 years of age.^22^

Each mammography session has a cost of £54.32 (US $69.53)^23^ and results in a quality-adjusted life-year (QALY) loss of 0.0014 due to associated discomfort.^24^ A summary table of the diagnostic accuracy of mammography is presented in eTable 1 in Supplement 1. Sensitivity estimates are sourced from recent estimates in a population-based screening program and vary based on breast density, ranging from 62% (dense) to 90% (not dense).^25^ False-positive results occur when no underlying tumor is present during a screening, with chances ranging from 1.5% (not dense) to 2.9% (dense),^25^ and they cause a QALY loss of 0.0771.^26^ Mammographic results indicating cancer leads to further assessments, which verify whether the cancer is present, and involve mammography, ultrasonography, and biopsy totaling £484.90 (US $620.67).^27^ Detailed breakdowns of mammography’s diagnostic accuracy, screening and further assessment costs, and screen-related QALY losses are found in eTables 2 and 3 in Supplement 1.

#### Model Event: Interval Between Screening Appointments

Between screening appointments, cancers are detected immediately in primary care on reaching the age of symptomatic detection, with the primary care appointment costing £37.00 (US $47.36),^28^ and further assessment totaling £484.90 (US $620.67) NHS cost.^27^ Mortality due to other causes than breast cancer can occur and aging results in a health utility loss based on a published formula.^29^

#### Model Event: Cancer Treatment and Survival

After cancer detection, cancer is classified as ductal carcinoma in situ (DCIS) or invasive tumor TNM stages I to IV, determined by age and mode of detection (at a screening appointment or symptomatically in primary care) using UK population screening data.^30^ The stage distribution is adjusted based on the time since the last screen, using US data comparing stage distribution by screening frequency.^31^ Detailed methodology and sources can be found in Hill et al.^32^

Treatment-related health losses^33^ and NHS costs^34,35^ vary by age, stage, mode of detection (screen or symptomatic), and time since detection (for follow-up costs and health recovery). Health utility losses after cancer are taken from multivariate regression, using the utility decrements on age, stage, and mode of detection.^33^ Recovery times are 11 years for screen-detected and interval cancers and 12 years for symptomatic cancers, based on peak health-related quality of life values post cancer detection.^33^ Stage 4 cancers are assumed to show no improvement in quality of life.^26^

Cancer survival estimates come from Office for National Statistics mortality statistics^17,36^ and multivariable regressions.^37,38,39^ Mortality hazard ratios by stage^37^ and detection mode^38,39^ are applied to age-based mortality^17^ to determine life expectancy. Cancer treatment-related utility losses, costs, and survival estimates are available in eTables 4 to 6 in Supplement 1.

### Economic and Clinical Outcomes

Costs were assessed from a UK payer perspective^13^ and reported in 2022 pounds sterling.^27,28^ Quality-adjusted life-years and costs were discounted at 3.5%.^13^ The main economic outcome is incremental net monetary benefit (INMB) per woman, which quantifies in monetary terms the net benefit of interventions by reflecting the potential alternative use of intervention resources for other health care treatments.^40^ Incremental net monetary benefit is established from differences in patient costs, and from assigning a monetary value to the difference in QALYs, which we assume to be £20 000 (US $25 600), £30 000 (US $38 400), and £1 (US $1.28) per QALY. The latter represents a scenario where a decision-maker is reluctant to spend additional NHS resources to increase population health. Population-wide INMB is derived by multiplying the per-woman INMB by the population size of women at 50 years of age eligible for screening invitations, which is 174 523, sourced from the 2022 national breast screening program data.^20^ Clinical outcomes include tumor stage at detection, cancers detected during screening, breast cancer deaths prevented by RSBCRs in the population (174 523 women), and the number of screens conducted.

### Statistical Analysis

All analyses were performed using R, version 4.2.2 (R Program for Statistical Computing).^15^ We conducted external validation of the model against targets derived from the 2022 national breast screening program data (eMethods 2 and eTable 7 in Supplement 1).^30,41^ To identify where savings occur, cost results are divided into screening-related costs and those for treating DCIS and invasive cancers. Cancer treatment costs are broken down by stage (DCIS, stages I-II, and stages III-IV) and mode of detection (screen-detected and interval cancers). Deterministic sensitivity analysis is conducted by adjusting the cancer treatment and screening costs, health-related quality of life losses from cancer and screening, and mammography sensitivity. Probabilistic sensitivity analysis is performed using 250 Monte Carlo simulations^42^ using probabilistic sensitivity analysis parameter distributions reported in eTable 8 in Supplement 1. The probability that each RSBCR is cost-effective is illustrated on a cost-effectiveness acceptability curve.^40^ Population-wide INMB estimates from the 250 Monte Carlo simulations are calculated across cost per QALY values ranging from £1 (US $1.28) to £100 000 (US $128 000).

## Results

### Base-Case Results

Table 1 shows base-case economic results and the annual impact of the economic results in the entire population. All the AI-based regimens were associated with reduced NHS costs and increased QALYs compared with the current screening program. The strategy of conducting screening every 6 years for low risk, every 2 to 3 years for medium risk, and annually for high risk had the highest additional net monetary gain per woman invited for screening. This amounts to £346 (US $442.88) and £489 (US $625.92) under the assumption of QALY values being £20 000 (US $25 600) and £30 000 (US $38 400), respectively. Consequently, this leads to an annual net monetary benefit within the NHS screening program totaling £10.6 million (US $13.6 million) for QALY values of £1, £60.4 million (US $77.3 million) for QALY values of £20 000, and £85.3 million (US $109.2 million) for QALY values of £30 000. The 3 alternative approaches had comparable figures for the net monetary benefit gained. For instance, the screening strategy of 6 yearly, triannual, and biannual screening has the smallest incremental net monetary benefit, amounting to £188 (US $240.64) and £236 (US $302.08) per person, assuming QALY values of £20 000 and £30 000, respectively. This resulted in an annual population incremental net benefit of £32.7 million (US $41.9 million), assuming £20 000 per QALY and £41.2 million (US $52.7 million), assuming £30 000 per QALY.

**Table 1.** Base-Case Results

Screening intervals for RSBCRs, y	Discounted outcomes		Incremental outcomes		Mean (SD) INMB per woman invited to screening at cost per QALY^a^^,^^b^		
	QALYs	Costs, £^b^	QALYs	Costs, £^b^	£20 000	£30 000	£1
3 (Current screening program)	16.476	1931	NA	NA	NA	NA	NA
1, 2, 3 or 6 y	16.490	1870	0.014	−60.72	346.0 (60.4)	489.0 (85.3)	61.0 (10.6)
1, 3, or 6 y	16.489	1865	0.014	−66.38	337.0 (58.7)	472.0 (82.3)	66.0 (11.6)
2, 3, or 6 y	16.481	1840	0.005	−90.55	188.0 (32.7)	236.0 (41.2)	91.0 (15.8
1, 3, or 4 y	16.490	1933	0.014	2.36	285.0 (49.8)	429.0 (74.8)	−2.0 (−0.4)

Abbreviations: INMB, incremental net monetary benefit; NA, not available; QALYs, quality-adjusted life-years; RSBCR, risk-stratified breast cancer screening regimen.

^a^ Expressed in millions of pounds sterling across the entire eligible screening population.

^b^ To convert pounds sterling to US dollars, multiply by 1.28.

Table 2 shows the clinical and screening program results. The clinical outcomes of all regimens showed an improvement compared with the current screening program. Compared with the current screening program, conducting screening every 6 years for low risk, every 2 to 3 years for medium risk, and annually for high risk resulted in a higher number of screen-detected cancers (10 549 vs 8943), a greater percentage of DCIS cancers at detection (17.1% vs 13.6%), a reduction in the number of screens (mean [SD], 3.22 [0.02] vs 4.68 [0.05]), and prevention of 834 deaths due to breast cancer.

**Table 2.** Clinical and Screening Program Results

Screening intervals for RSBCRs, y	Cancers detected by stage, No. (%)			Cancer deaths avoided, No. (%)	Screen-detected cancers, No. (%)	No. of screens per woman from first invitation to screening at age 50 y to last at age 70 y, mean (SD)
	DCIS	Stages I-II	Stages III-IV			
3 (Current screening program)	2330 (13.6)	12 847 (75.0)	1946 (11.4)	NA	8943 (52.2)	4.68 (0.05)
1, 2, 3 or 6	2926 (17.1)	12 529 (73.2)	1669 (9.7)	834 (4.9)	10 549 (61.6)	3.22 (0.01)
1, 3, or 6	2849 (16.6)	12 609 (73.6)	1666 (9.7)	776 (4.5)	10 543 (61.6)	3.21 (0.02)
2, 3, or 6	2525 (14.7)	12 707 (74.2)	1892 (11.0)	299 (1.7)	9567 (55.9)	3.06 (0.01)
1, 3, or 4	3024 (17.7)	12 456 (72.7)	1644 (9.6)	899 (5.2)	10 825 (63.2)	4.33 (0.02)

Abbreviations: DCIS, ductal carcinoma in situ; NA, not available; RSBCR, risk-stratified breast cancer screening regimen.

### Deterministic Sensitivity Analysis Results

The deterministic sensitivity analysis results (eTables 9-19 in Supplement 1) align with the cost-effectiveness of screening regimens found in the base-case model. Screening every 6 years for low risk, every 2 to 3 years for medium risk, and annually for high risk was likely to be most beneficial. Risk-stratified breast cancer screening regimens maintained their cost-effectiveness compared with the current program and each other. The breakdown of NHS costs incurred by women (eTable 20 in Supplement 1) show screening costs are lower in the RSBCR than in the current program. Cancer treatment costs incurred are larger for RSBCRs due to life extension for patients with cancer from increased screen detection of cancers and early cancer detection (see Table 2).

### Probabilistic Sensitivity Analysis Results

**Figure 2.**
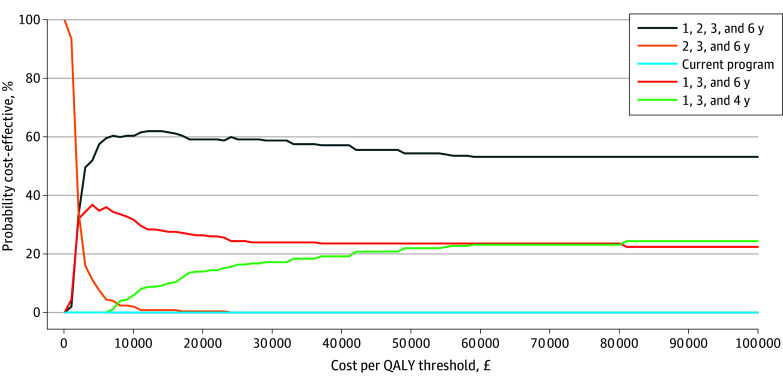
Cost-Effectiveness Acceptability Curve QALY indicates quality-adjusted life-year. To convert pounds sterling to US dollars, multiply by 1.28.

The probabilistic sensitivity analysis results in Table 3 demonstrate an improvement in the cost-effectiveness of screening regimens compared with the base-case model, without a shift in the ranking of programs from most to least cost-effective. The regimen with screening intervals of 1, 2, 3, or 6 years had the highest probability of being cost-effective (59% at £20 000 [US $25 600] per QALY) and had the largest net monetary benefit for all cost per QALY thresholds (eFigure in Supplement 1). The cost-effectiveness acceptability curve (Figure 2) shows the regimen of 2, 3, or 6 years was likely to be the least cost-effective alternative option for RSBCRs, and the current screening program has a negligible probability of being cost-effective across all cost per QALY thresholds.

**Table 3.** Probabilistic Sensitivity Analysis Results

Screening intervals for RSBCRs, y	Discounted outcomes		Mean (SD) INMB per woman invited to screening, £^a^^,^^b^			Probability most cost-effective at cost per QALY, %^b^		
	QALYs	Costs, £^b^	At £20 000 cost per QALY^b^	At £30 000 cost per QALY	At £1 cost per QALY	£20 000	£30 000	£1
3 (Current screening program)	16.440	1961	NA	NA	NA	0	0	0
1, 2, 3 or 6	16.500	1939	1263.0 (220.4)	1884.0 (328.8)	20.0 (3.5)	59	59	0
1, 3, or 6	16.497	1935	1209.0 (211.1)	1802.0 (314.5)	25.0 (4.3)	26	24	0
2, 3, or 6	16.466	1879	632.0 (110.3)	909.0 (158.7)	77.0 (13.4)	0	0	100
1, 3, or 4	16.498	2001	1137.0 (198.4)	1725.0 (301.1)	−40.0 (−6.9)	14	17	0

Abbreviations: INMB, incremental net monetary benefit; NA, not available; QALY, quality-adjusted life-years; RSBCR, risk-stratified breast cancer screening regimen.

^a^ Expressed in millions of pounds sterling across the entire eligible screening population.

^b^ To convert pounds sterling to US dollars, multiply by 1.28.

## Discussion

Under our analytic model, AI-based risk-stratified screening is likely to be cost-effective compared with the current one-size-fits-all screening program. Our findings are consistent with previous modeling studies demonstrating the cost-effectiveness of risk-stratified screening, whether based on traditional risk factors like family history or newer methods such as polygenic risk scores.^26,43,44,45^ However, our model is the first to suggest that health care resources might be reduced with RSBCRs, while attaining at least the same effectiveness for the population.^43,44,45^ This study is also the first to assess the cost-effectiveness of AI interpretation of breast images for risk assessment during routine screening.^43,44,45^ This might be more feasible at scale than the other risk assessment methods.

While the estimated incremental economic benefits per individual invited to breast screening may seem modest in the base-case model, ranging from £188 (US $240.64) to £346 (US $442.88), the annual monetary gains for the NHS might be substantial, estimated to range from £32.7 million (US $ 41.9 million) to £60.4 million (US $77.3 million). In the probabilistic model, these savings are around 3 times larger. The health economic model suggests that RSBCRs can reduce screening utilization, NHS costs, and invasive cancer in the population and increase QALYs. The reduction in the number of screenings with an RSBCR could free up resources to address screening backlogs and reduce wait times where those problems exist in the UK,^46^ potentially further improving breast cancer outcomes.

Annual screening RSBCRs lead to higher percentages of screen-detected cancers and greater percentages of DCIS cancers at detection, along with higher QALYs. However, biennial screening for the highest-risk group, with corresponding 3 and 6 yearly screening for other risk groups, incurs lower costs due to fewer screenings. Screen utilization is not the only explanation of the difference in cost-effectiveness among the regimens. Other contributing factors are the proportion of individuals at medium and high risk within the population undergoing intensified screening surveillance, their uptake to screening appointments, and the accuracy of mammography in women with denser breast tissue that makes cancers harder to detect. Although cost-effectiveness is important, it is not the only factor to consider when comparing the benefits of the regimen.^47^ For example, a population accustomed to the UK’s triennial screening program may view a 6-year gap between screenings as unacceptable, favoring a 4-year interval instead. This change to an RSBCR screening program with a 4- year screening interval for patients at low risk would generate an annual monetary benefit of £50 million (US $64 million), assuming a QALY is valued at £20 000 (US $25 600). Significant variations in screening frequency within programs can be accepted by patients. In the UK, colonoscopy surveillance guidelines for cancer vary by risk level, with no surveillance for patients at low risk, every 3 years for patients at intermediate risk, and annually for patients at high risk.^48^

### Limitations

This study has some limitations. First, the model is parametrized for the UK. Findings may not be directly generalizable to alternative geographical populations, such as the US or elsewhere in Europe. Clinical guidelines in these regions often recommend universal annual or biennial screening, while a triennial screening program is in place in the UK, and typically screening also begins at earlier ages in these regions.^49^ Therefore, the comparator screen detection rates based on triennial screening, on which the model depends, are likely to differ across populations with varied screening frequencies and onset ages. For example, in a setting where annual screening is the norm, all the regimens considered would reduce costs but also effectiveness. Further analysis using the model could usefully contribute to decisions on the economic value of decreasing intensity of screening based on breast cancer AI in such settings.

## Conclusions

In this decision analytical model study of risk-based screening with AI-based risk assessment, risk-based screening delivered health benefits while using fewer NHS resources compared with the current UK breast screening program. New studies to prospectively evaluate AI-guided screening appear warranted.

## Notes

Supplement 1. **eMethods 1.** Detailed Explanation of Method of Assigning Breast Density and Mirai Scores **eTable 1.** Summary of Breast Density-Based Values of the Diagnostic Accuracy of Mammography and Annual Change in Breast Density **eTable 2.** Summary of Screening Costs and Sources **eTable 3.** Health Utility and QALY Parameters **eTable 4.** Hazard Ratios for Invasive Cancer Survival **eTable 5.** Costs and Invasive Cancer Care by Age, Mode of Detection, and Duration Since Detection **eTable 6.** Costs of DCIS Cancer Care by Age, Mode of Detection, and Duration Since Detection **eMethods 2.** External Validation of the Model **eTable 7.** Validation Results and Actual NHS Screening Outcomes Compared With Model Predictions **eTable 8.** PSA Parameter Table **eTable 9.** Disutility Related to Screening Increased by 20% **eTable 10.** Disutility Related to Screening Decreased by 20% **eTable 11.** Mammographic Screen Sensitivity Increased by 20% **eTable 12.** Mammographic Screen Sensitivity Decreased by 20% **eTable 13.** Cancer Treatment Costs Increased by 20% **eTable 14.** Cancer Treatment Costs Decreased by 20% **eTable 15.** Mammogram and Further Assessment Costs Increased by 20% **eTable 16.** Mammogram and Further Assessment Costs Decreased by 20% **eTable 17.** Health-Related Quality of Life Loss From Cancer Increased by 20% **eTable 18.** Health-Related Quality of Life Loss From Cancer Decreased by 20% **eTable 19.** Screen Detection Has No Independent Effect on Cancer Mortality **eTable 20.** Breakdown of NHS Incurred **eFigure.** Incremental Population Net Monetary Benefit
eReferences

Supplement 2.
Data Sharing Statement
