# Supplementary material for: Cost-Effectiveness of AI for Risk-Stratified Breast Cancer Screening
Source: JAMA Netw Open. 2024 Sep 5;7(9):e2431715. doi: 10.1001/jamanetworkopen.2024.31715 (PMC11377997; doi:10.1001/jamanetworkopen.2024.31715)
Supplement: Supplement 1. — eMethods 1. Detailed Explanation of Method of Assigning Breast Density and Mirai Scores eTable 1. Summary of Breast Density-Based Values of the Diagnostic Accuracy of Mammography and Annual Change in Breast Density eTable 2. Summary of Screening Costs and Sources eTable 3. Health Utility and QALY Parameters eTable 4. Hazard Ratios for Invasive Cancer Survival eTable 5. Costs and Invasive Cancer Care by Age, Mode of Detection, and Duration Since Detection eTable 6. Costs of DCIS Cancer Care by Age, Mode of Detection, and Duration Since Detection eMethods 2. External Validation of the Model eTable 7. Validation Results and Actual NHS Screening Outcomes Compared With Model Predictions eTable 8. PSA Parameter Table eTable 9. Disutility Related to Screening Increased by 20% eTable 10. Disutility Related to Screening Decreased by 20% eTable 11. Mammographic Screen Sensitivity Increased by 20% eTable 12. Mammographic Screen Sensitivity Decreased by 20% eTable 13. Cancer Treatment Costs Increased by 20% eTable 14. Cancer Treatment Costs Decreased by 20% eTable 15. Mammogram and Further Assessment Costs Increased by 20% eTable 16. Mammogram and Further Assessment Costs Decreased by 20% eTable 17. Health-Related Quality of Life Loss From Cancer Increased by 20% eTable 18. Health-Related Quality of Life Loss From Cancer Decreased by 20% eTable 19. Screen Detection Has No Independent Effect on Cancer Mortality eTable 20. Breakdown of NHS Incurred eFigure. Incremental Population Net Monetary Benefit eReferences [file jamanetwopen-e2431715-s001.pdf]

Supplementary Online Content

Hill H, Roadevin C, Duffy S, Mandrik O, Brentnall A. Cost-effectiveness of AI for risk-stratified breast cancer screening. *JAMA Netw Open*. 2024;7(9):e2431715. doi:10.1001/jamanetworkopen.2024.31715

eMethods 1. Detailed Explanation of Method of Assigning Breast Density and Mirai Scores
eTable 1. Summary of Breast Density-Based Values of the Diagnostic Accuracy of Mammography and Annual Change in Breast Density
eTable 2. Summary of Screening Costs and Sources
eTable 3. Health Utility and QALY Parameters
eTable 4. Hazard Ratios for Invasive Cancer Survival
eTable 5. Costs and Invasive Cancer Care by Age, Mode of Detection, and Duration Since Detection
eTable 6. Costs of DCIS Cancer Care by Age, Mode of Detection, and Duration Since Detection
eMethods 2. External Validation of the Model
eTable 7. Validation Results and Actual NHS Screening Outcomes Compared With Model Predictions
eTable 8. PSA Parameter Table
eTable 9. Disutility Related to Screening Increased by 20%
eTable 10. Disutility Related to Screening Decreased by 20%
eTable 11. Mammographic Screen Sensitivity Increased by 20%
eTable 12. Mammographic Screen Sensitivity Decreased by 20%
eTable 13. Cancer Treatment Costs Increased by 20%
eTable 14. Cancer Treatment Costs Decreased by 20%
eTable 15. Mammogram and Further Assessment Costs Increased by 20%
eTable 16. Mammogram and Further Assessment Costs Decreased by 20%
eTable 17. Health-Related Quality of Life Loss From Cancer Increased by 20%
eTable 18. Health-Related Quality of Life Loss From Cancer Decreased by 20%
eTable 19. Screen Detection Has No Independent Effect on Cancer Mortality
eTable 20. Breakdown of NHS Incurred
eFigure. Incremental Population Net Monetary Benefit
eReferences.

This supplementary material has been provided by the authors to give readers additional information about their work.

© 2024 Hill H et al. *JAMA Network Open*.

eMethods 1. Detailed Explanation of Method of Assigning Breast Density and Mirai Scores

Volpara Breast density is initially determined by an individual's age at their first screening appointment and the presence of cancer at that time, specifically whether the age falls after the onset of cancer tumour genesis. The annual change in Volpara breast density, taken from a recent study[1], depends upon the presence of cancer and the classification of breast density as high (VDG3 & VDG4) or low (VDG3 & VDG4). The change is a decrease of 0.157% (in the absence of cancer and with high breast density), 0.137% (in the presence of cancer and with high breast density), 0.057% (in the absence of cancer and with low breast density), and 0.033% (in the presence of cancer and with low breast density).

Initial risk score is assigned at the first screening appointment based on the individual's age and whether they will ever have a cancer tumour. This aligns with the categorisation of AI risk scores found in the Mirai case-control data which the risk score distribution is taken from. Subsequent resampling occurs during follow-up screenings for women who have never had a cancer tumour. Although independent resampling can result in risk fluctuations at the individual level, these fluctuations remained minimal due to the relatively narrow confidence intervals around the mean risk scores within the five-year age groups, and it incorporates the full uncertainty in risk scores over time at the population-level. Of note is that the correlation between risk scores and breast density is still present for women without cancer at a population via age-based sampling of both risk scores and breast density. However, the relationship (correlation) between breast density and risk scores only has an influence on economic outcomes (NHS costs and QALYs) for women with cancer, owing to both factors being influences on the likelihood of detecting cancer. In the case of risk scores, this impact is manifested through more frequent screening for women at high risk which increases likelihood of cancer detection, while higher breast density lowers mammographic screen sensitivity, lowering the likelihood of successful cancer detection. Therefore, this relationship is not an influence on economic outcomes for women without cancer, as the cancer detection probability is zero. In summary, the independent re-sampling of risk scores approach captures the complete uncertainty in risk scores over time at the population level, and the evaluation is conducted at the population level.

For women who develop cancer, a different approach is taken. This is because the AI risk score data indicates a significant increase in their assessed risk during the screening conducted three years prior to cancer detection, compared to women of the same age without cancer. Incorporating this information into the model poses a challenge in an economic simulation because risk scores must be assigned before cancer detection (as risk scores determine the type of screening program received the individual). That is, the model simulation can not assign risk scores to match the Mirai data because in the model there can be no guarantee that cancer will be detected three years after the abrupt increase begins. To incorporate into the model the abrupt increase in risk scores three years before cancer detection, even without certainty in the model about whether cancer will detected at the three year, the following approach of anchoring the change in risk to an age in which cancer would likely be detected (rather than age at which it is detected, as per the Mirai data). An age at which cancer would likely be detected is established for each individual with cancer over their lifetime by sampling from a normal distribution lead time, based on parameters taken from a recent study[2] (a mean of 2.09 years and 95% confidence interval: 2.07 to 2.12). Lead time is then subtracted from the age of symptomatic detection of cancer. With the resulting age at which cancer would likely be detected. Since the change in risk score data spans a

© 2024 Hill H et al. *JAMA Network Open*.

three-year period, the model subsequently subtracts three years from this new reference point. This revised reference point is where we incorporate the abrupt increase in risk scores for women with cancer. The change in risk from the revised reference point is sample by age from the change in risk scores three years prior to cancer detection in the AI risk score data. For consistency with the method used to establish risk scores on and after the revised reference point, the risk scores for ages earlier than the age of revised reference point are determined by modifying the risk score calculated at preceding screening appointment. This adjustment is made based on the annual age-related risk change for women without a history of cancer, as derived from the Mirai dataset.
The AI risk score data had information on change in risk three years prior to cancer detection. However, it is possible that the rapid increase in risk score may have begun earlier than three years (i.e. in the period where the Mirai data is censored). To account for this possibility, in sensitivity analysis, we consider this new reference point as the time at which Tumour visibility occurs in model, calculated as the age at which cancer is symptomatically detected minus the tumour presence time.

eTable 1. Summary of Breast Density-Based Values of the Diagnostic Accuracy of Mammography and Annual Change in Breast Density

Variable	Values
**Sensitivity**	
VDG1 (less than 3.5% Volpara percentage breast density)	90%
VDG2 (3.5–7.4%)	82%
VDG3 (7.5–15.5%)	71%
VDG4 (greater than 15.5%)	62%
**False positive chance in women without cancer**	
VDG1	1.5%
VDG2	2.2%
VDG3	2.9%
VDG4	2.8%
**Annual decline in breast density**	
VDG1 and VDG2, with the absence of cancer	0.057%
VDG3 and VDG4, with the absence of cancer	0.157%
VDG1 and VDG2, with cancer	0.033%
VDG3 and VDG4, with cancer	0.137%

eTable 2. Summary of Screening Costs and Sources

Cost parameter changes	Unit cost	Source and costing assumption
Cost of mammography	£54.32	Cost (£54.32) was calculated using the most recent mammography data available (NHS reference costs 2005/06, £40), adjusted exclusively using the NHS Cost Inflation Index, up to the year 2021/22.
Cost of further assessment	£484.90	Sum of mammography, ultrasound and biopsy cost
Cost of ultrasound (utilised as a component of further assessment costs following the positive detection of cancer).	£106.01	2020/2021 tariff cost for Ultrasound Scan, Mobile, or Intraoperative Procedures, lasting less than 20 minutes (HRG code: RD44Z)
Biopsy cost at further assessment updated	£324.70	In the Breast Screening Program England 2021-22 report (Page 24), it is reported that 50.13% of women undergoing further assessment have a core biopsy (NHS BSP), and 1.29% are reported to undergo an open biopsy.<br>The assumed cost for a core biopsy is calculated as the weighted average (weighted by activity, as

© 2024 Hill H et al. *JAMA Network Open*.

		reported in 2021/2022 NHS reference costs) of two core breast surgery outpatient procedures NHS reference costs (2021/22): Core Needle Biopsy of Lesion of Breast and Associated Lymph Nodes (YJ03Z: £485.73) and Core Needle Biopsy of Axillary Lymph Nodes (YJ04Z: £726.51), resulting in a total cost of £618.93.<br>For an open biopsy, the assumed cost is the sum of the core biopsy cost and the cost of Insertion of Wire or Marker (2021/22 tariff costs, code YJ10Z: £422.27). The prevalence adjusted cost of biopsies is £324.70
The cost of inviting individuals to a screening and determining the risk score solely through breast density-based risk assessment methods.	£14.52.	The projected unit cost for a breast screening invitation was £8.37 in 1999/2000 prices. After adjusting for inflation to 2021/22 using the NHS Cost Inflation Index, it is £14.52.
Cost for attending a general practitioner for symptomatically detected cancer.	£37.00	Excluding direct care staff costs, the cost related to GPs are calculated based on the unit costs for a session lasting 9.22 minutes (2021/22) unit costs

eTable 3. Health Utility and QALY Parameters

**Health utility and QALY decrements**	**DCIS and Stage 1**	**Stage 2**	**Stage 3**	**Stage 4**
Health utility decrements after cancer detection by duration of time from detection				
In year 1	0.098	0.128	0.138	0.158
Annual change from year 1 to year 11 for screen detected cancer	0.0088	0.0118	0.0128	0.0148
Annual change from year 1 to year 12 for symptomatic detected cancer	0.0089	0.0116	0.0125	0.158
Health-related quality of life improvement in cancer recovery for individuals with at least one screen attendance	0.01			
QALY loss from a mammogram screen	0.0014			
QALY loss from False positive mammogram screen	0.0771			
Annual age-related health utility loss	Based on the following formulae from Ara and Brazier (2010):<br>0.0002587 multiplied by age,<br>0.0000332 multiplied by age squared			

eTable 4. Hazard Ratios for Invasive Cancer Survival

**Cancer survival risks**	**Hazard Ratio**
TNM stage 1	1 (general population survival)
TNM stage 2	3.39
TNM stage 3	11.96
TNM stage 4	30.05
Age at cancer detection	
50-69	1
70-74	1.49
75-79	1.36
80+	1.23
Screen detected compared to interval cancers	0.7142
screen-detected compared to individuals who have never attended screenings	0.649

© 2024 Hill H et al. *JAMA Network Open*.

eTable 5. Costs and Invasive Cancer Care by Age, Mode of Detection, and Duration Since Detection

**Year after cancer detection**	**TNM Stage 1–2**					**TNM Stage 3–4**				
**Screen detected cancer:**	**Ages 18–60**	**Ages 60-69**	**Ages 70-79**	**Ages 80-89**	**Ages 90**	**Ages 18–60**	**Ages 60-69**	**Ages 70-79**	**Ages 80-89**	**Ages 90 or over**
Year 1	£11,257	£10,059	£8,643	£5,899	£3,445	£14,048	£12,564	£10,082	£6,902	£4,059
Year 2	£3,517	£3,143	£2,877	£1,964	£11,47.30	£7,563	£6,764	£4,844	£3,316	£1,950
Year 3	£2,046	£1,828	£2,452	£1,674	£9,77.84	£4,944	£4,422	£4,207	£2,880	£1,694
Year 4	£1,704	£1,523	£2,537	£1,731	£10,11.52	£3,832	£3,428	£3,881	£2,657	£1,562
Year 5	£1,694	£1,514	£2,363	£1,613	£942	£3,713	£3,322	£3,924	£2,686	£1,580
Year 6	£1,621	£1,448	£2,474	£1,688	£986	£3,458	£3,092	£3,693	£2,528	£1,487
Year 7	£1,460	£1,305	£2,347	£1,602	£936	£3,422	£3,061	£3,852	£2,638	£1,550
Year 8	£1,441	£1,288	£2,427	£1,657	£967	£3,345	£2,991	£3,257	£2,229	£1,312
Year 9 to 11	£1,340	£1,197	£2,507	£1,711	£1,000	£2,416	£2,161	£3,891	£2,664	£1,566
**Symptom detected cancer:**										
Year 1	£11,673	£10,475	£9,058	£6,314	£3,861	£14,463	£12,979	£10,497	£7,317	£4,474
Year 2	£3,646	£3,272	£3,016	£2,102	£1,285	£7,786	£6,987	£5,044	£3,516	£2,149
Year 3	£2,122	£1,904	£2,570	£1,793	£1,096	£5,090	£4,568	£4,381	£3,054	£1,867
Year 4	£1,767	£1,586	£2,659	£1,854	£1,134	£3,946	£3,542	£4,041	£2,817	£1,722

© 2024 Hill H et al. *JAMA Network Open*.

Year after cancer detection	TNM Stage 1–2					TNM Stage 3–4				
Year 5	£1,757	£1,577	£2,477	£1,726	£1,056	£3,824	£3,431	£4,086	£2,848	£1,742
Year 6	£1,681	£1,508	£2,592	£1,807	£1,105	£3,560	£3,195	£3,846	£2,681	£1,639
Year 7	£1,515	£1,359	£2,460	£1,715	£1,048	£3,524	£3,162	£4,011	£2,797	£1,709
Year 8	£1,495	£1,341	£2,544	£1,774	£1,084	£3,444	£3,091	£3,391	£2,364	£1,445
Year 9 to 12	£1,389	£1,247	£2,628	£1,831	£1,120	£2,487	£2,233	£4,051	£2,824	£1,727

eTable 6. Costs of DCIS Cancer Care by Age, Mode of Detection, and Duration Since Detection

Year after cancer detection	Screen detected					Symptomatically detected				
	Ages 18–60	Ages 60-69	Ages 70-79	Ages 80-89	Ages 90	Ages 18–60	Ages 60-69	Ages 70-79	Ages 80-89	Ages 90 or over
1	£8,312	£7,428	£6,382	£4,356	£2,544	£8,620	£7,735	£6,689	£4,663	£2,851
2	£2,568	£2,296	£2,102	£1,435	£838	£2,664	£2,390	£2,203	£1,536	£939
3	£1,495	£1,336	£1,791	£1,223	£714	£1,549	£1,390	£1,878	£1,309	£800
4	£1,245	£1,113	£1,853	£1,265	£739	£1,290	£1,159	£1,942	£1,354	£828
5	£1,237	£1,105	£1,726	£1,178	£688	£1,283	£1,152	£1,809	£1,261	£772
6	£1,184	£1,058	£1,806	£1,233	£720	£1,227	£1,101	£1,894	£1,320	£807
7	£1,066	£954	£1,715	£1,170	£683	£1,106	£993	£1,797	£1,253	£766
8	£1,053	£941	£1,774	£1,210	£707	£1,092	£980	£1,859	£1,296	£793
9-11	£979	£875	£1,831	£1,250	£730	£1,015	£910	£1,920	£1,338	£818
12	£0	£0	£0	£0	£0	£1,015	£910	£1,920	£1,338	£818

eMethods 2. External Validation of the Model

One validation target is the percentage of tumours that are DCIS upon cancer detection (12.77%). This information is taken from Table A4.3 in the National Audit of Breast Cancer in Older Patients 2022 annual report[4]. The absence of details on the TNM stage of invasive cancer at the time of detection in this report restricts the validation to distinguishing between DCIS and invasive cancer. A validation target is the percentage of screen detected cancer. This is based on the count of cancers diagnosed at a screening appointment in comparison to all other pathways to diagnosis found in Table A4.2 in the same report. The model includes parameters for uptake, but the average number of screenings a woman undergoes is not specified due to its dependence on the following factors: age at screening invitation, a woman's history of attending screening appointments, and whether it is her initial or preventive screening invitation. The validation target is set at 4.74 screening appointments based on the following calculation. Table 1 in the Breast Screening Program data report from England 2018-19[4], shows a 71.08% uptake in 2019. Women aged between 50 and 70 receive invitations for screening every three years, which is an average of 6.67 invitations. When this number is multiplied by the uptake rate the result is an average attendance of 4.74 screening appointments.

eTable 7. Validation Results and Actual NHS Screening Outcomes Compared With Model Predictions

© 2024 Hill H et al. *JAMA Network Open*.

Validation target	Validation target outcomes	Model prediction	Source if target outcomes
Percentage of tumours that are DCIS upon detection	13.8%	13.6%	National Audit of Breast Cancer in Older Patients 2022 report[3]
Percentage of screen detected cancers	55.8%	52.2%	National Audit of Breast Cancer in Older Patients 2022 report[3]
Number of screening appointments attended	4.74	4.68	Women aged between 50 and 70 receive invitations for screening every three years, totalling 6.67 invitations on average. When combined with the 2019 uptake rate[4], this is an average of 4.74 attended screening appointments.

eTable 8. PSA Parameter Table

Table of PSA parameter values

Unless otherwise stated, all distributions assumed that the standard error was 20% of the mean.

Variable	Distribution	Mean value
Breast density change as women age	Standard normal	Supplemental Table 1
QALY loss for experiencing mammography screening	Truncated Normal (standard error 0.0144)[5]	Supplemental Table 3:
QALY loss for experiencing FP	Truncated Normal	Supplemental Table 3:
Excess risk of mortality 10 years post DCIS diagnosis	Log-normal	Table14 in Hill et al., 2022[9]
Relative mortality rates for invasive cancer	Normal, truncated to be >1 with uncertainty based on confidence intervals in reference[6-8]	Supplemental Table 4.
Relative mortality rates for cancer due to screen attendance history	Standard Normal distribution	Supplemental Table 4.
Tumour presence time	Sampled from linear model	
Invasive cancer utility decrements by stage and time since diagnosis	Gamma	Supplemental Table 3
Utility gain for screen detected cancers	Normal distribution	Supplemental Table 3
Screening costs	Gamma	Supplemental Table 2
Cost of treating cancer	Gamma	Supplemental Table 5 & 6.
Digital mammography sensitivity	Beta (VDG1 (A 265, B21; VDG2 (A1680, B381);	Supplemental Table 1

© 2024 Hill H et al. *JAMA Network Open*.

	VDG3 (A 966, B402);<br>VDG4 (A 214; B131)	
False-positive rates for instruments	Standard normal	Supplemental Table 1

Deterministic sensitivity analysis results

eTable 9. Disutility Related to Screening Increased by 20%

**Screening intervals (years) for risk stratified screening regimens**	**Discounted outcomes**		**Incremental outcomes**		**Incremental net monetary benefit** per woman invited to screening	
	QALYs	Costs (£)	QALYs	Costs (£)	At £20,000 cost per QALY	At £30,000 cost per QALY
Current screening program (3y)	16.474	1931	-	-	-	-
1, 2, 3 or 6y	16.489	1870	0.015	- 61	360	509
1, 3, or 6y	16.488	1865	0.014	- 66	350	493
2, 3, or 6y	16.479	1840	0.006	- 91	203	259
1, 3, or 4y	16.488	1933	0.015	2	290	437

eTable 10. Disutility Related to Screening Decreased by 20%

**Screening intervals (years) for risk stratified screening regimens**	**Discounted outcomes**		**Incremental outcomes**		**Incremental net monetary benefit** per woman invited to screening	
	QALYs	Costs (£)	QALYs	Costs (£)	At £20,000 cost per QALY	At £30,000 cost per QALY
Current screening program (3y)	16.478	1931				
1, 2, 3 or 6y	16.492	1870	0.014	- 61	332	468
1, 3, or 6y	16.491	1865	0.013	- 66	323	451
2, 3, or 6y	16.482	1840	0.004	- 91	172	213
1, 3, or 4y	16.492	1933	0.014	2	280	422

© 2024 Hill H et al. *JAMA Network Open*.

eTable 11. Mammographic Screen Sensitivity Increased by 20%

**Screening intervals (years) for risk stratified screening regimens**	**Discounted outcomes**		**Incremental outcomes**		**Incremental net monetary benefit** per woman invited to screening	
	QALYs	Costs (£)	QALYs	Costs (£)	At £20,000 cost per QALY	At £30,000 cost per QALY
Current screening program (3y)	16.480	1950	-	-	-	-
1, 2, 3 or 6y	16.494	1882	0.015	- 68	361	508
1, 3, or 6y	16.493	1881	0.013	- 69	336	470
2, 3, or 6y	16.489	1853	0.009	- 97	279	370
1, 3, or 4y	16.493	1942	0.014	-8	282	420

eTable 12. Mammographic Screen Sensitivity Decreased by 20%

**Screening intervals (years) for risk stratified screening regimens**	**Discounted outcomes**		**Incremental outcomes**		**Incremental net monetary benefit** per woman invited to screening	
	QALYs	Costs (£)	QALYs	Costs (£)	At £20,000 cost per QALY	At £30,000 cost per QALY
Current screening program (3y)	16.466	1917	-	-	-	-
1, 2, 3 or 6y	16.483	1853	0.018	- 64	417	593
1, 3, or 6y	16.484	1857	0.018	- 60	420	600
2, 3, or 6y	16.474	1821	0.009	- 95	266	352
1, 3, or 4y	16.483	1917	0.017	0	343	515

eTable 13. Cancer Treatment Costs Increased by 20%

**Screening intervals (years) for risk stratified screening regimens**	**Discounted outcomes**		**Incremental outcomes**		**Incremental net monetary benefit** per woman invited to screening	
	QALYs	Costs (£)	QALYs	Costs (£)	At £20,000 cost per QALY	At £30,000 cost per QALY
Current screening program (3y)	16.476	2211	-	-	-	-
1, 2, 3 or 6y	16.490	2149	0.014	- 62	347	490
1, 3, or 6y	16.489	2144	0.014	- 67	337	472
2, 3, or 6y	16.481	2122	0.005	- 89	186	235
1, 3, or 4y	16.490	2211	0.014	0	288	432

© 2024 Hill H et al. *JAMA Network Open*.

eTable 14. Cancer Treatment Costs Decreased by 20%

**Screening intervals (years) for risk stratified screening regimens**	**Discounted outcomes**		**Incremental outcomes**		**Incremental net monetary benefit** per woman invited to screening	
	QALYs	Costs (£)	QALYs	Costs (£)	At £20,000 cost per QALY	At £30,000 cost per QALY
Current screening program (3y)	16.476	1651	-	-	-	-
1, 2, 3 or 6y	16.490	1591	0.014	- 60	345	488
1, 3, or 6y	16.489	1585	0.014	- 66	336	471
2, 3, or 6y	16.481	1559	0.005	- 92	189	237
1, 3, or 4y	16.490	1656	0.014	5	283	427

eTable 15. Mammogram and Further Assessment Costs Increased by 20%

**Screening intervals (years) for risk stratified screening regimens**	**Discounted outcomes**		**Incremental outcomes**		**Incremental net monetary benefit** per woman invited to screening	
	QALYs	Costs (£)	QALYs	Costs (£)	At £20,000 cost per QALY	At £30,000 cost per QALY
Current screening program (3y)	16.476	2000	-	-	-	-
1, 2, 3 or 6y	16.490	1920	0.014	- 80	365	508
1, 3, or 6y	16.489	1914	0.014	- 86	356	491
2, 3, or 6y	16.481	1888	0.005	- 112	209	257
1, 3, or 4y	16.490	1995	0.014	- 5	292	436

eTable 16. Mammogram and Further Assessment Costs Decreased by 20%

**Screening intervals (years) for risk stratified screening regimens**	**Discounted outcomes**		**Incremental outcomes**		**Incremental net monetary benefit** per woman invited to screening	
	QALYs	Costs (£)	QALYs	Costs (£)	At £20,000 cost per QALY	At £30,000 cost per QALY
Current screening program (3y)	16.476	1862	-	-	-	-
1, 2, 3 or 6y	16.490	1821	0.014	- 42	327	469
1, 3, or 6y	16.489	1815	0.014	- 47	317	452
2, 3, or 6y	16.481	1793	0.005	- 69	166	215
1, 3, or 4y	16.490	1871	0.014	9	278	422

© 2024 Hill H et al. *JAMA Network Open*.

eTable 17. Health-Related Quality of Life Loss From Cancer Increased by 20%

Screening intervals (years) for risk stratified screening regimens	Discounted outcomes		Incremental outcomes		Incremental net monetary benefit per woman invited to screening	
	QALYs	Costs (£)	QALYs	Costs (£)	At £20,000 cost per QALY	At £30,000 cost per QALY
Current screening program (3y)	16.470	1931	-	-	-	-
1, 2, 3 or 6y	16.484	1870	0.014	- 61	343	484
1, 3, or 6y	16.483	1865	0.013	- 66	334	468
2, 3, or 6y	16.475	1840	0.005	- 91	186	233
1, 3, or 4y	16.484	1933	0.014	2	281	423

eTable 18. Health-Related Quality of Life Loss From Cancer Decreased by 20%

Screening intervals (years) for risk stratified screening regimens	Discounted outcomes		Incremental outcomes		Incremental net monetary benefit per woman invited to screening	
	QALYs	Costs (£)	QALYs	Costs (£)	At £20,000 cost per QALY	At £30,000 cost per QALY
Current screening program (3y)	16.482	1931	-	-	-	-
1, 2, 3 or 6y	16.496	1870	0.014	- 61	349	494
1, 3, or 6y	16.496	1865	0.014	- 66	339	476
2, 3, or 6y	16.487	1840	0.005	- 91	189	239
1, 3, or 4y	16.497	1933	0.015	2	289	435

eTable 19. Screen Detection Has No Independent Effect on Cancer Mortality

Screening intervals (years) for risk stratified screening regimens	Discounted outcomes		Incremental outcomes		Incremental net monetary benefit per woman invited to screening	
	QALYs	Costs (£)	QALYs	Costs (£)	At £20,000 cost per QALY	At £30,000 cost per QALY
Current screening program (3y)	16.477	1934	-	-	-	-
1, 2, 3 or 6y	16.491	1873	0.014	- 61	345	487
1, 3, or 6y	16.490	1867	0.013	- 66	335	470
2, 3, or 6y	16.482	1843	0.005	- 90	189	238
1, 3, or 4y	16.491	1936	0.014	3	286	431

eTable 20. Breakdown of NHS Incurred

© 2024 Hill H et al. *JAMA Network Open*.

**Screening intervals (years) for RSBCRs**	Total Cost (£)	Screening cost (£)	Cancer treatment cost (£)	Invasive treatment cost (£)	DCIS treatment cost (£)	Costs of cancer treatment (£) across different cancer types and subgroups			
						Stage 1/2	Stage 3/4	Screen detected	Interval cancers
Current screening program	1970	385	1585	1399	186	1597	1765	1804	1346
1, 2, 3 or 6y	1959	338	1621	1395	226	1643	1973	1725	1453
1, 3, or 6y	1953	337	1616	1397	219	1645	1909	1725	1441
2, 3, or 6y	1894	293	1601	1405	196	1613	1885	1756	1406
1, 3, or 4y	2020	397	1623	1387	236	1650	1945	1738	1425

eFigure. Incremental Population Net Monetary Benefit

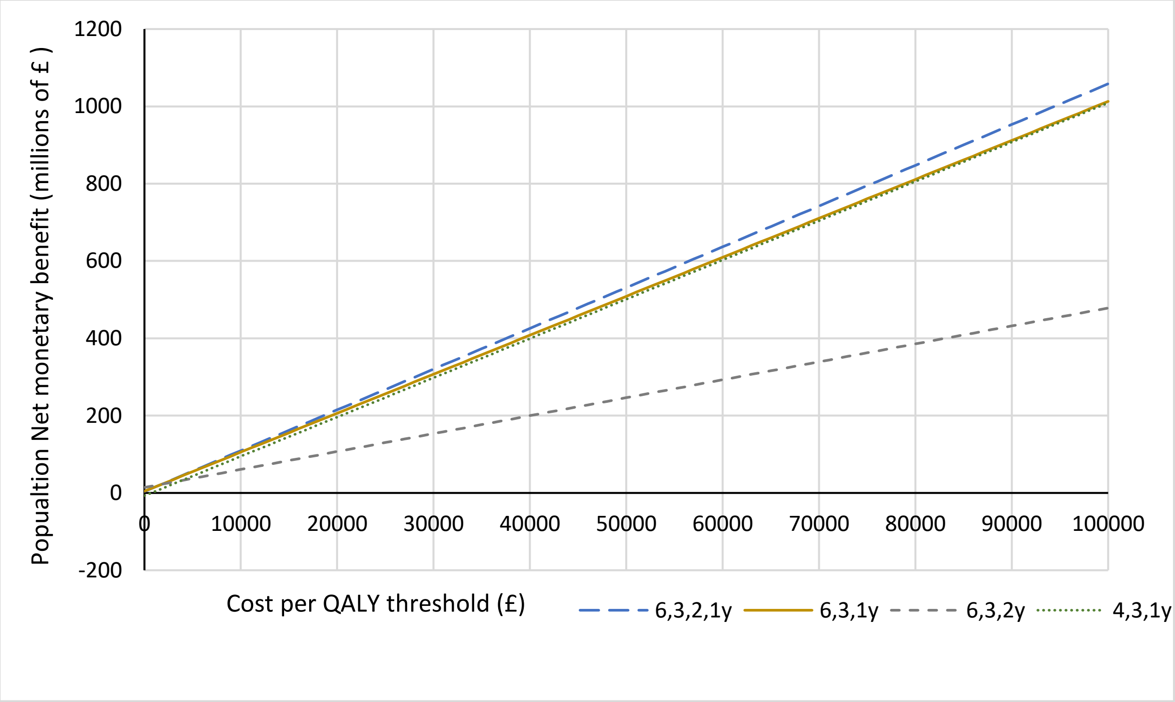

eReferences.

1. Brandt, K.R., Scott, C.G., Miglioretti, D.L., Jensen, M.R., Mahmoudzadeh, A.P., Hruska, C., Ma, L., Wu, F.F., Cummings, S.R., Norman, A.D. and Engmann, N.J., 2019. Automated volumetric breast density measures: differential change between breasts in women with and without breast cancer. *Breast Cancer Research*, *21*(1), pp.1-10.
2. Abrahamsson, L., Isheden, G., Czene, K. and Humphreys, K., 2020. Continuous tumour growth models, lead time estimation and length bias in breast cancer screening studies. *Statistical Methods in Medical Research*, *29*(2), pp.374-395
3. Royal College of Surgeons of England. National Audit of Breast Cancer in Older Patients 2022.

© 2024 Hill H et al. *JAMA Network Open*.

4. NHS England. NHS Breast Screening Programme, England 2019-20. National statistics, Official statistics. 2021 Available at: https://digital.nhs.uk/data-and-information/publications/statistical/breast-screening-programme/england---2021-22
5. Li, L., Severens, J. L., & Mandrik, O. Disutility associated with cancer screening programs: a systematic review. *PloS one* **14**(7), e0220148 (2019).
6. (37) Ali AM, Greenberg D, Wishart GC, Pharoah P. Patient and tumour characteristics, management, and age-specific survival in women with breast cancer in the East of England. *British journal of cancer*. 2011 Feb;104(4):564-70.
7. (38) Puvanesarajah S, Gapstur SM, Patel AV, Sherman ME, Flanders WD, Gansler T, Troester MA, Gaudet MM. Mode of detection and breast cancer mortality by follow-up time and tumor characteristics among screened women in Cancer Prevention Study-II. *Breast Cancer Research and Treatment*. 2019 Oct;177:679-89.
8. (39) Nagtegaal, I.D., Allgood, P.C., Duffy, S.W., Kearins, O., Sullivan, E.O., Tappenden, N., Wallis, M. and Lawrence, G., 2011. Prognosis and pathology of screen-detected carcinomas: How different are they?. *Cancer,* 117(7), pp.1360-1368.
9. Hill H, Kearns B, Duffy S, Pashaya N, Sasieni P, Offman J (2022) Estimating the cost-effectiveness of risk stratified breast cancer screening in the UK. Policy Research Unit in Economic Evaluation of Health and Social Care Interventions. Universities of Sheffield and York. Report 066. DOI: https://doi.org/10.15131/shef.data.25219262

© 2024 Hill H et al. *JAMA Network Open*.
