# Supplementary material for: Cost-Effectiveness of AI for Risk-Stratified Breast Cancer Screening
Source: JAMA Netw Open. 2024 Sep 5;7(9):e2431715. doi: 10.1001/jamanetworkopen.2024.31715 (PMC11377997; doi:10.1001/jamanetworkopen.2024.31715)
Supplement: Supplement 2. — Data Sharing Statement [file jamanetwopen-e2431715-s002.pdf]

Data Sharing Statement

Hill. An Economic Evaluation of Use of Artificial Intelligence for Risk-Stratified Breast Cancer Screening. *JAMA Netw Open*. Published September 05, 2024.
doi:10.1001/jamanetworkopen.2024.31715

Data
Data available: No

Additional Information
Explanation for why data not available: Not applicable. No data was collected in this study. The data used in the model is from published studies.
